# Flash Communication: Rhodium Complexes of Acetamide-Derived PAlP Pincer

**DOI:** 10.1021/acs.organomet.4c00351

**Published:** 2024-09-19

**Authors:** R. Noah Sladek, Nattamai Bhuvanesh, Oleg V. Ozerov

**Affiliations:** Department of Chemistry, Texas A&M University, College Station, Texas 77842, United States

## Abstract

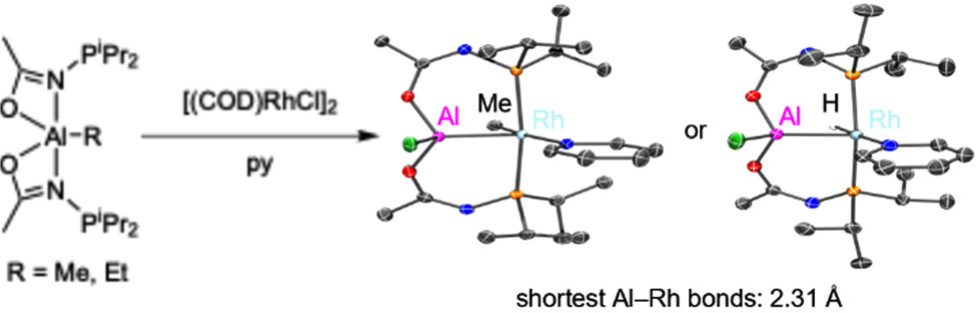

The new alane/bis(phosphine) PAlP pincer-type ligands **2-Me** and **2-Et** have been prepared by protolysis reactions of *N*-(diisopropylphoshino)acetamide with Me_3_Al or Et_3_Al in a 2:1 ratio. Upon reaction with a “RhCl” source and pyridine, **2-Me** gave rise to a (PAlP)RhMe(py) compound (**4-Me**), while **2-Et** led to the analogous hydride complex (PAlP)RhH(py) (**4-H**), with a migration of the chloride from Rh to Al. **4-Me** and **4-H** possess the shortest Rh–Al bonds to date.

Pincer-style tridentate ligands bearing an aluminum-based central moiety have recently attracted considerable attention due to their versatile coordination behavior and unique electronic properties. Complexes containing an aluminate (**A**, Figure 1),^1^ aluminyl (**B**–**D**),^2−4^ and alane (**E**, **F**)^5,6^ have been described. The coordination flexibility of Al is further evident from the fact that the aluminyls **B**–**D** come with three-, five-, or six-coordinate Al, and compounds of the **F** type can convert the aluminyl (X-type ligand) moiety to an alane (Z-type ligand)^7^ via exchange of substituents between Al and the transition metal (TM).^6^ Computational studies have explored the unusual TM^δ−^–Al^δ+^ polarity involved with the aluminyl ligand, which is ascribed to the lower electronegativity of Al.^8−12^ Aluminyl-supported Rh complexes have been used by the Nakao group as catalysts for heterocycle C–H functionalization and cleavage of Ar–F and Ar–O bonds.^3,13−16^

**Figure 1:**
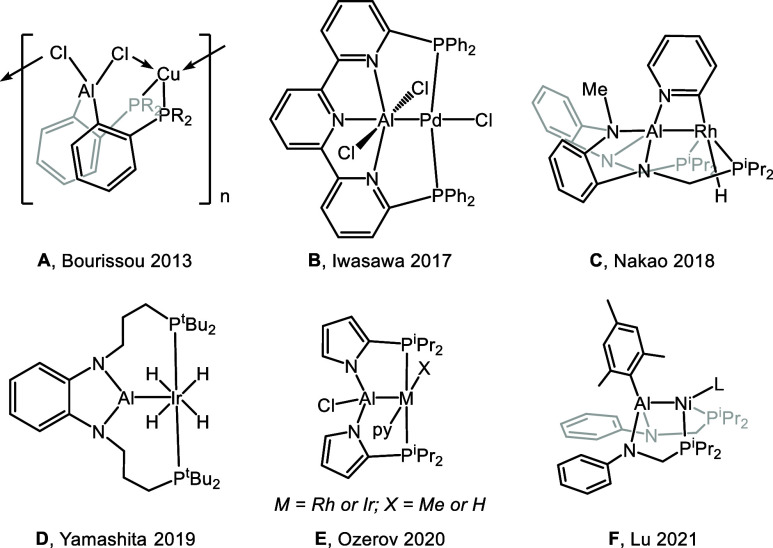
Selected examples of pincer complexes with aluminum central moieties (**A**–**F**).

Our group previously reported on the Rh and Ir complexes of the **E** type, which displayed short TM–Al distances and facile migration of hydride, methyl, and chloride substituents between Al and TM. Type **E** complexes can be viewed as 5,5-pincers based on the Fryzuk notation^17^ because they form two five-membered metal-containing rings. We were interested in exploring analogous 6,6-PAlP structures, because they may permit more geometric flexibility needed to accommodate tripodal binding. In addition, we wished to explore an even higher Lewis acid strength of the central alane. In this work, we report a successful execution of this notion using Braunstein’s *N*-(diisopropylphoshino)acetamide^18^ (**1**, Figure 2) as a convenient building block in place of 2-phosphinopyrrole used to construct **E**.^5,19^ We chose the acetamide linker in part because a wide variety of carboxamides are readily available for potential future tuning of the properties of the targeted ligand type.

**Figure 2:**
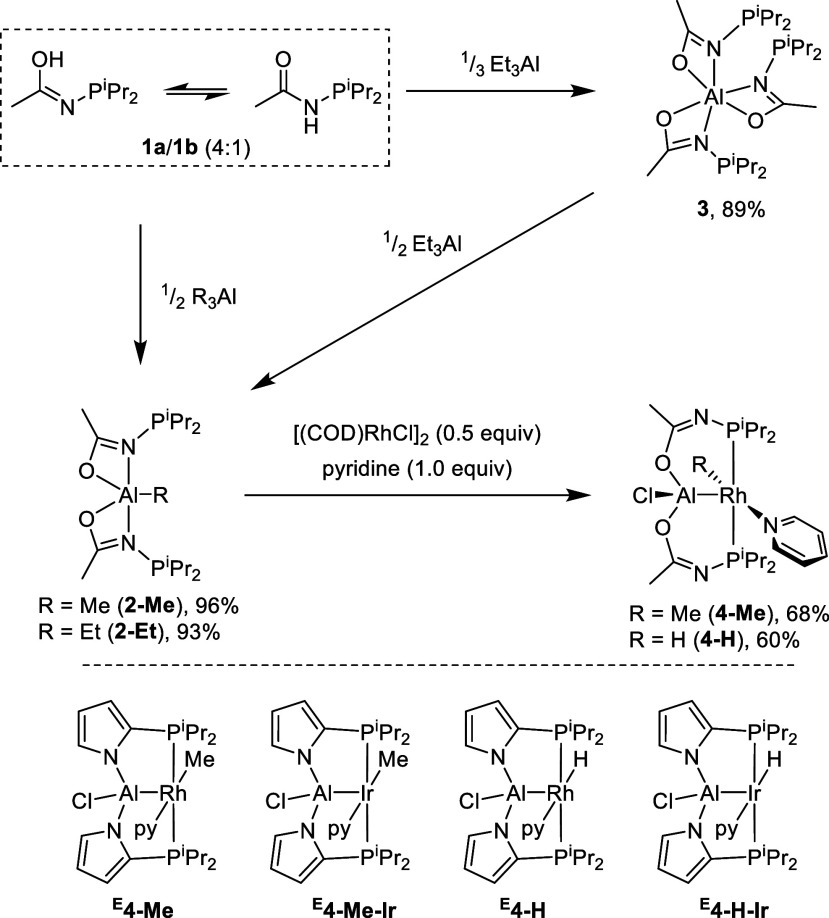
Synthesis of acetamide-derived ligands and pincer complexes of rhodium and selected previously reported compounds (bottom).

Compound **1** exists as a mixture of tautomers **1a**/**1b**.^18^ Reaction of 2 equiv of **1** with either Me_3_Al or Et_3_Al proceeded cleanly to give the compounds **2-Me** and **2-Et**, respectively, with presumed loss of methane or ethane. **2-Me** and **2-Et** were obtained as oils of apparent >95% purity by NMR analysis and were suitable for further reactions. If a deficiency of R_3_Al is used, then another product (**3**) can be seen. It was isolated in high yield as a crystalline solid from the protolysis of **1** with Et_3_Al in a 3:1 ratio. Isolated **3** was reacted with 0.5 equiv of Et_3_Al to cleanly generate **2-Et**, indicating that the imidate and alkyl substituents exchange freely at ambient temperature. All three compounds **2-Me**, **2-Et**, and **3** give rise to a single ^31^P NMR resonance (δ 66.1, 66.1, and 71.6 ppm, respectively), as well as a single acetamide Me resonance in the ^1^H and ^13^C NMR spectra. However, the isopropyl resonances in the ^1^H NMR spectrum of **3** are sharp and indicate only one kind of isopropyl environment, whereas in the spectra of **2-Me**/**2-Et**, the isopropyl resonances are broad and correspond to two isopropyl environments. The solid-state structure of **3** (Figure S1) revealed the bidentate (κ^2^-*N,O*) coordination of three imidate groups to Al with no Al–P interactions. The solid-state structure is not 3-fold symmetric, so some exchange process is implied in solution. It is likely that bidentate (κ^2^-*N,O*) coordination is also present in **2-Me**/**2-Et**, and ostensibly, the exchange process is slower, resulting in lower symmetry. In addition, it is not clear whether any Al–P interactions or bridging N or O atoms might occur in **2-Me**/**2-Et**, as well.

Treatment of **2-Me** or **2-Et** with pyridine followed by 0.5 equiv of [(COD)RhCl]_2_ resulted in the formation of new compounds **4-Me** and **4-H**, isolated in good yields upon workup. ^1^H NMR data are consistent with a *C*_*s*_ symmetry at ambient temperature and support the presence of the Rh–Me functionality in **4-Me** (δ 0.52 ppm, 3H, td, *J*_H–P_ = 7 Hz, *J*_H–Rh_ = 2 Hz), and of Rh–H in **4-H** (δ −17.30 ppm, 1H, dt, *J*_H–Rh_ = 23 Hz, *J*_H–P_ = 19 Hz). These values are quite similar to those observed previously for complexes of the type **E** (Figure 3). The formation of Rh–H bonds out of **2-Et** proceeds via transfer of the Et group to Rh, followed by irreversible β-hydrogen elimination with release of ethylene (observed in situ).

**Figure 3:**
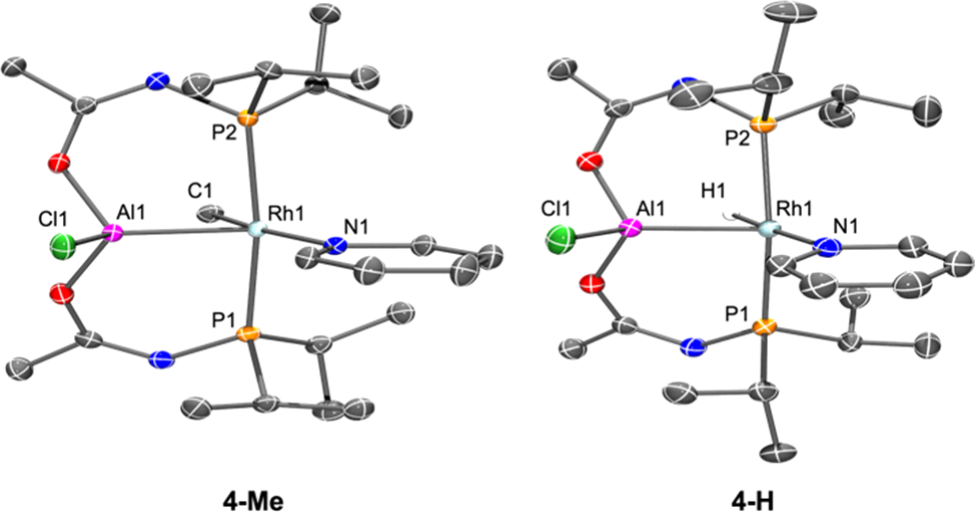
ORTEP drawings (50% probability ellipsoids) of **4-Me** and **4-H** displaying selected labeling. The toluene molecule in **4-H** and hydrogen atoms (except for Rh−H) are omitted for clarity. Selected bond distances (Å) and angles (deg) for **4-Me**: Rh1–Al1, 2.3058(8); Rh1–C1, 2.100(3); Rh1–N1, 2.146(2); Al1–Cl1, 2.1680(13); Rh1–P1, 2.3257(7); Rh1–P2, 2.3320(7); P1–Rh1–P2, 168.81(3); C1–Rh1–N1, 168.59(10); Al1–Rh1–N1, 111.23(6). Selected bond distances (Å) and angles (deg) for **4-H**: Rh1–Al1, 2.3062(8); Rh1–N1, 2.1526(18); Al1–Cl1, 2.1703 (8); Rh1–P1, 2.3168(7); Rh1–P2, 2.3028(7); P1–Rh1–P2, 163.00(3); H1–Rh1–N1, 177.18(6); Cl1–Al1–Rh1, 113.89(3); Al1–Rh1–N1, 118.42(5).

X-ray diffractometry studies on single crystals of **4-Me** and **4-H** confirmed the structures expected from solution NMR data (Figure 3), with pyridine coordinated to Rh and chloride coordinated to Al, oriented *anti* to Rh–Me or Rh–H. The pyridine ring is sandwiched between a pair of isopopropyl groups, but the conformation of the latter is different in the structure of **4-Me** vs **4-H**. In **4-Me**, the centroid of the pyridine ring is closer to the isopropyl Me group, but in **4-H**, it is closer to the isopropyl CH. Remarkably, this conformational difference can also be seen in complexes of type **E**. In the ^1^H NMR spectra, the presence of pyridine with its ring-current effect shifts a pair of Me groups upfield. We previously noted that this shift was more pronounced for ^**E**^**4-Me**/^**E**^**4-Me-Ir** than for ^**E**^**4-H**/^**E**^**4-H-Ir** (Figure 2), and we observe the same disparity for **4-Me** vs **4-H**: the most upfield Me resonance for **4-Me** is at 0.52 ppm, but it is at 1.08 ppm for **4-H**. Lastly, the isopropyl groups restrict rotation about the Rh–N bond. In **4-Me**, the rotation about the Rh–N bond is apparently slower than in **4-H**, as evidenced by the presence of five distinct ^1^H NMR resonances for the pyridine hydrogens in **4-Me**, but only three (in 2:1:2 ratio) for **4-H**. This is again similar to the contrast between ^**E**^**4-Me**/^**E**^**4-Me-Ir** (five pyridine resonances) vs for ^**E**^**4-H**/^**E**^**4-H-Ir** (three broadened pyridine resonances). The structural explanation for these phenomena is that the Rh–Me functionality is larger than Rh–H and exerts greater steric repulsion, felt by the −P^i^Pr_2_ groups. As a result, the P–Rh–P angle opens up to a greater degree in **4-Me** (ca. 169°) vs **4-H** (ca. 164°) and the isopropyl groups are pushed closer to the pyridine ring.

The Rh–Al distances in **4-Me**/**4-H** are essentially the same at about 2.31 Å. They are shorter than those in **E** (2.33–2.34 Å) and appear to be the shortest Rh–Al bonds to date, albeit only by a small margin.^20,21^ The slight difference vs **E** may arise from the acetamide oxygen rendering the Al center in **4-Me**/**4-H** a stronger Lewis acid than the bis(N-pyrrolyl)aluminum center in **E**. The larger Al/Rh ring size in **4-Me**/**4-H** may also permit more geometric flexibility. We previously noted that theoretical analysis indicated a modest degree of interaction between Al–H and Rh/Ir in complexes ^**E**^**4-H**. Given the similarity of structures, this is likely to be the case here (Al–H distances are similar, in the 2.1–2.2 Å range). A meaningful Rh–Me to Al interaction is less likely given the large C−Al separation of ca. 2.8 Å in **4-Me**.

In summary, we have been able to prepare Rh complexes of a PAlP pincer with an expanded ring system based on an acetamide building block. The oxygens of the acetamide linker confer a high level of Lewis acidity on the central alane moiety. It can be easily envisioned that an array of related ligands could be synthesized by using different carboxamide building blocks.

## Supporting Information Available

Details of synthetic procedures and X-ray diffractrometry, graphical NMR spectra, and other spectroscopic characterization (PDF)
