# Supplementary material for: Flash Communication: Rhodium Complexes of Acetamide-Derived PAlP Pincer
Source: Organometallics. 2024 Sep 19;43(19):2169–71. doi: 10.1021/acs.organomet.4c00351 (PMC11481167; doi:10.1021/acs.organomet.4c00351)
Supplement: Supplementary file 1 — om4c00351_si_001.pdf [file om4c00351_si_001.pdf]

Supporting Information

Flash Communication: Rhodium Complexes of Acetamide-Derived PAlP Pincer

R. Noah Sladek, Nattamai S. Bhuvanesh, and Oleg V. Ozerov*

Department of Chemistry, Texas A&M University, College Station, TX 77842, USA
E-mail: ozerov@chem.tamu.edu.

Table of Contents

I. General Considerations **S3**

II. Experimental Procedures **S4**

III. X-Ray Structural Determination Details **S8**

V. NMR Spectra **S12**

VI. SI References **S29**

I. General Considerations

Unless otherwise specified, all manipulations were performed either inside an argon-filled glove box, or by using Schlenk techniques. Toluene and THF were dried using a PureSolv MD-5 Solvent Purification System and were stored over 4Å molecular sieves in an argon-filled glove box. C_6D_6 and pyridine were dried over CaH_2, distilled or vacuum transferred, and stored in an argon-filled glovebox over 4Å molecular sieves prior to use. *N*-[bis(diisopropyl)phosphino]acetamide and $[Rh(COD)Cl]_2$ were synthesized according to literature precedent.[1,2] All other chemicals were used as received from commercial vendors. Argon was used from standard gas cylinders with 99.998% purity. All NMR spectra were acquired on a Bruker 400 (^{1}H NMR, 400.09 MHz; ^{13}C NMR, 100.60 MHz; ^{31}P NMR, 161.96 MHz) and Bruker 500 (^{1}H NMR, 499.703 MHz; ^{13}C NMR, 125.697 MHz; ^{31}P NMR, 202.265 MHz) spectrometer. All ^{1}H and ^{13}C NMR spectra were referenced internally to the residual solvent signal (C_6D_6 at δ 7.16 for ^{1}H and δ 128.06 for ^{13}C NMR). All ^{31}P NMR spectra were externally referenced to an 85% phosphoric acid solution δ 0. Elemental analysis was performed by Robertson Microlit Laboratories, Ledgewood NJ.

II. Experimental Procedures

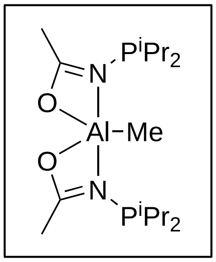

Synthesis of 2-Me. In a 50 mL Schlenk flask, trimethylaluminum (715 µL, 1.43 mmol, 2.0 M in toluene) was added dropwise to a solution of *N*-[bis(diisopropyl)phosphino]acetamide (500 mg, 2.85 mmol) in 10 mL toluene. The solution gradually changed color from clear to pale-yellow. After stirring the mixture at room temperature for 16 h, volatiles were removed *in vacuo* to afford a clear oil (535 mg, 96%) that was judged to be 95+% pure by NMR spectroscopy. ^{1}H NMR (400 MHz, C_6D_6): δ 2.22 (m, 2H, C*H*Me$_2$), 2.10 (d, $J_{P–H}$ = 1.6 Hz, 6H OC(*Me*)=N), 1.82 (m, 2H, C*H*Me$_2$), 1.20 (m, 6H, CH*Me*$_2$), 0.99 (m, 18H, CH*Me*$_2$), -0.10 (s, 3H, Al*Me*). ^{31}P{^{1}H} NMR (162 MHz, C_6D_6): δ 66.1 (s). ^{13}C{^{1}H} NMR (101 MHz, C_6D_6): δ 190.9 (d, $J_{C–P}$ = 24.3 Hz, O*C*(Me)=N), 26.5 (br, *C*HMe$_2$), 25.2 (br, *C*HMe$_2$), 18.7 (m, OC(*Me*)=N & CH*Me*$_2$), 17.5 (s, CH*Me*$_2$), -10.1 (br, Al–*Me*).

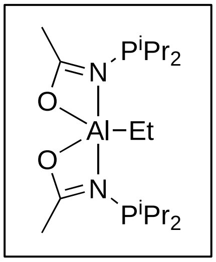

Synthesis of 2-Et. In a 50 mL Schlenk flask, triethylaluminum (1.43 mL, 1.43 mmol, 1.0 M in heptane) was added dropwise to a solution of *N*-[bis(diisopropyl)phosphino]acetamide (500 mg, 2.85 mmol) in 10 mL toluene. The solution gradually changed color from clear to pale-yellow. After stirring the mixture at room temperature for 16 h, volatiles were removed *in vacuo* to afford a clear oil (551 mg, 93%) that was judged to be 95+% pure by NMR spectroscopy. ^{1}H NMR (400 MHz, C_6D_6): δ 2.19 (m, 2H, C*H*Me$_2$), 2.10 (d, $J_{H–P}$ = 1.8 Hz, 6H, *Me*C(=N)O), 1.80 (m, 2H, C*H*Me$_2$), 1.43 (t, $J_{H–H}$ = 8.2 Hz, 3H, Al–CH$_2$C*H$_3$*) 1.20 (br, 6H, CH*Me*$_2$), 0.98 (br, 18H, CH*Me*$_2$) 0.53 (br, 2H, Al–C*H$_2$*CH$_3$). ^{31}P{^{1}H} NMR (162 MHz, C_6D_6): δ 66.1 (s). ^{13}C{^{1}H} NMR (101 MHz, C_6D_6): δ 191.1 (d, $J_{C–P}$ = 24.4 Hz, Me*C*(=N)O), 26.5 (br, *C*HMe$_2$), 25.1 (br, *C*HMe$_2$), 19.4 – 18.0 (m, *Me*C(=N)O & CH*Me*$_2$), 17.4 (br, CH*Me*$_2$), 9.8 (s, Al–CH$_2$*C*H$_3$), 0.6 (br, Al–*C*H$_2$CH$_3$).

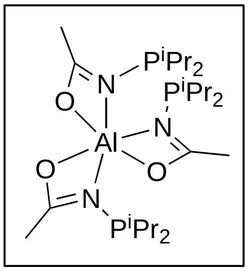

Synthesis of 3. In a 50 mL Schlenk flask, triethylaluminum (0.95 mL, 0.95 mmol, 1.0 M in heptane) was added dropwise to a solution of *N*-[bis(diisopropyl)phosphino]acetamide (500 mg, 2.85 mmol) in 10 mL toluene. After stirring the mixture at room temperature for 16 h, volatiles were removed *in vacuo*. The resulting white solid was recrystallized from pentane, yielding colorless crystals (464 mg, 89%). ^{1}H NMR (500 MHz, C_6D_6): δ 2.16 (s, 3H, *Me*C(=N)O), 2.00 (septd, $J_{H–P}$ = 7.1 Hz, $J_{H–H}$ = 3.5 Hz, 2H, C*H*Me$_2$), 1.24 (dd, $J_{H–P}$ = 13.8 Hz, $J_{H–H}$ = 7.1 Hz, 6H, CH*Me$_2$*), 1.12 (dd, $J_{H–P}$ = 13.8 Hz, $J_{H–H}$ = 7.1 Hz, 6H, CH*Me*$_2$). ^{31}P{^{1}H} NMR (202 MHz, C_6D_6): δ 71.6 (s). ^{13}C{^{1}H} NMR (126 MHz, C_6D_6): 189.1 (d, $J_{C–P}$ = 24.4 Hz, Me*C*(=N)O), 26.9 (d, $J_{C–P}$ = 12.9 Hz, *C*HMe$_2$), 20.0 (d, $J_{C–P}$ = 16.2 Hz, CH*Me*$_2$), 19.5 (d, $J_{C–P}$ = 19.2 Hz, *Me*C(=N)O), 18.8 (d, $J_{C–P}$ = 17.5 Hz, CH*Me*$_2$).

Comproportionation of 3 with Et$_3$Al. In a J. Young tube, triethylaluminum (10 µL, 0.010 mmol) was added to a 600 µL solution of **3** (11 mg, 0.020 mmol) in C_6D_6. The mixture was heated at 60 ˚C for 1 h. ^{31}P{^{1}H} NMR analysis of the mixture revealed formation of **2-Et** (Figure S10).

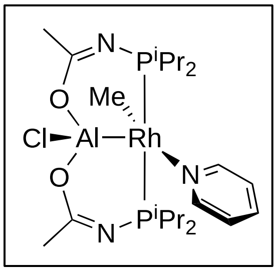

Synthesis of 4-Me. In a 50 mL Schlenk flask, **2-Me** (482 mg, 1.23 mmol) and pyridine (99 μL, 1.23 mmol) were dissolved in 10 mL toluene. $[(COD)RhCl]_2$ (304 mg, 0.617 mmol) was added while stirring. After stirring the mixture at room temperature for 24 h, volatiles were removed *in vacuo*. The resulting yellow solid was recrystallized from THF, yielding an off-white solid (440 mg, 68%). ^{1}H NMR (400 MHz, C_6D_6): δ 9.96 (d, 1H, $J_{H–H}$ = 5.5 Hz, *Pyridyl*), 8.42 (d, 1H, $J_{H–H}$ = 5.5 Hz, *Pyridyl*), 6.71 (m, 1H, *Pyridyl*), 6.57 (t, 1H, $J_{H–H}$ = 6.3 Hz, *Pyridyl*), 6.36 (t, 1H, $J_{H–H}$ = 6.6 Hz, *Pyridyl*), 2.23 (m, 2H, C*H*Me$_2$), 2.16 (s, 6H, *Me*C(=N)O), 1.77 (m, 2H, C*H*Me$_2$), 1.38 (q, $J_{H–H}$ = 7.5 Hz, 6H, CH*Me$_2$*), 1.24 (q, $J_{H–H}$ = 7.1 Hz, 6H, CH*Me$_2$*), 0.99 (q, $J_{H–H}$ = 7.0 Hz, 6H, CH*Me$_2$*), 0.64 (q, $J_{H–H}$ = 7.2 Hz, 6H, CH*Me$_2$*), 0.52 (td, $J_{H–P}$ = 6.7 Hz, $J_{H–Rh}$ = 1.7 Hz, 3H, Rh–*Me*). ^{31}P{^{1}H} NMR (162 MHz, C_6D_6): δ 65.4 (d, $J_{P–Rh}$ = 111.0 Hz). ^{13}C{^{1}H} NMR (101 MHz, C_6D_6): δ 171.7 (s, Me*C*(=N)O), 155.5 (s, s, *Pyridine*), 149.3 (s, *Pyridine*), 136.3 (s, *Pyridine*), 125.6 (s, *Pyridine*), 123.6 (s, *Pyridine*), 30.5 (t, $J_{P–C}$ = 17.1 Hz, CH*Me$_2$*), 28.2 (t, $J_{P–C}$ = 15.5 Hz, C*H*Me$_2$), 20.0 (t, $J_{P–C}$ = 3.07 Hz, CH*Me$_2$*), 18.3 (s, *Me*C(=N)O), 18.2 (t, $J_{P–C}$ = 1.6 Hz, CH*Me$_2$*), 17.8 (t, $J_{P–C}$ = 1.43 Hz, CH*Me$_2$*), -10.6 (dt, $J_{P–Rh}$ = 22.3 Hz, $J_{P–C}$ = 7.9 Hz, Rh–*Me*). Anal. Calcd for $C_{22}H_{42}AlClN_3O_2P_2Rh$: C, 43.47; H, 6.96; N, 6.91. Found: C, 43.41; H, 6.72; N, 6.78.

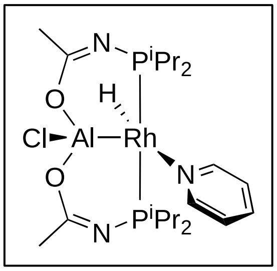

Synthesis of 4-H. In a 50 mL Schlenk flask, **2-Et** (275 mg, 0.68 mmol) and pyridine (55 μL, 0.68 mmol) were dissolved in 5 mL toluene. $[(COD)RhCl]_2$ (168 mg, 0.34 mmol) was added while stirring. After stirring the mixture at room temperature for 24 h, volatiles were removed *in vacuo*. The resulting yellow solid was recrystallized from THF, yielding an off-white solid (240 mg, 60%). ^{1}H NMR (500 MHz, C_6D_6): 8.98 (br, 2H, *Pyridyl*), 6.70 (t, $J_{H–H}$ = 7.7 Hz, 1H, *Pyridyl*), 6.47 (t, $J_{H–H}$ = 7.7 Hz, 2H, *Pyridyl*), 2.14 (m, 8H, C*H*Me$_2$ & *Me*C(=N)O), 1.41 (q, $J_{H–H}$ = 7.1 Hz,

6H, CH*Me*$_2$), 1.21 (m, 2H, C*H*Me$_2$), 1.06 (m, 18H, CH*Me*$_2$), -17.30 (dt, $J_{H–Rh}$ = 23.0 Hz, $J_{H–P}$ = 18.9 Hz, 1H, Rh–*H*). ^{31}P{^{1}H} NMR (202 MHz, C_6D_6): δ 68.6 (d, $J_{P–Rh}$ = 111.5 Hz). ^{13}C{^{1}H} NMR (126 MHz, C_6D_6): δ 173.9 (s, Me*C*(=N)O), 151.8 (s, *Pyridine*), 136.2 (s, *Pyridine*), 124.5 (s, *Pyridine*), 30.4 (t, $J_{P–C}$ = 19.6 Hz, *C*HMe$_2$), 27.6 (t, $J_{P–C}$ = 5.3 Hz, CH*Me*$_2$), 27.3 (t, $J_{P–C}$ = 15.6 Hz, *C*HMe$_2$), 20.1 (s, *Me*C(=N)O), 19.2 (t, $J_{P–C}$ = 5.3 Hz, CH*Me*$_2$), 18.6 (t, $J_{P–C}$ = 2.0 Hz, CH*Me*$_2$), 16.4 (s, CH*Me*$_2$).

***In situ* observation of ethylene.** In a J. Young tube, [(COD)RhCl]$_2$ (31 mg, 0.06 mmol) was added to a solution of **2-Et** (50 mg, 0.12 mmol) and pyridine (10 μL, 0.12 mmol) in 600 μL C_6D_6. The resulting mixture was stirred at room temperature for 24 h. ^{1}H NMR analysis revealed with presence of free ethylene (Figure S18).

III. X-Ray Structural Determination Details

X-Ray data collection, solution, and refinement for 3 (CCDC 2363382). A Leica M80 microscope was used to identify a suitable single colorless block-shaped crystal showing well defined faces with dimensions 0.50 × 0.40 × 0.26 mm^3 from a representative sample of crystals of the same habit. The crystal mounted on a nylon loop was then placed in a cold nitrogen stream (Oxford) maintained at T = 110 K. Crystal screening, unit cell determination, and data collection were carried out using a Bruker Quest (PHOTON III) diffractometer. Data was measured using ϕ and ω scans with MoK_α radiation. Data was collected to a maximum resolution of Θ = 27.520° (0.77 Å). The unit cell was refined using SAINT V8.38A on 9788 reflections, 19% of the observed reflections.[3] Integrated Intensity information for each reflection was obtained by reduction of data frames using SAINT V8.38A.[3] The final completeness is 99.90 % out to 27.520° in Θ. SADABS-2016/2 was used for absorption correction.[4] wR_2(int) was 0.1330 before and 0.0689 after correction. The ratio of minimum to maximum transmission is 0.8466. The $\lambda/2$ correction factor is not present. The absorption coefficient μ of this material is 0.241 mm^{-1} at this wavelength (λ = 0.71073 Å) and the minimum and maximum transmissions are 0.176 and 0.208. Systematic reflection conditions and statistical tests of the data suggested the space group $P2_1/c$ and was confirmed by ShelXT 2018/2 structure solution program using dual methods.[5] The structure was refined by full matrix least squares minimization on $\boldsymbol{F^2}$ using version 2018/3 of XL.[5] All non-hydrogen atoms were refined anisotropically. Hydrogen atom positions were calculated geometrically and refined using the riding model.

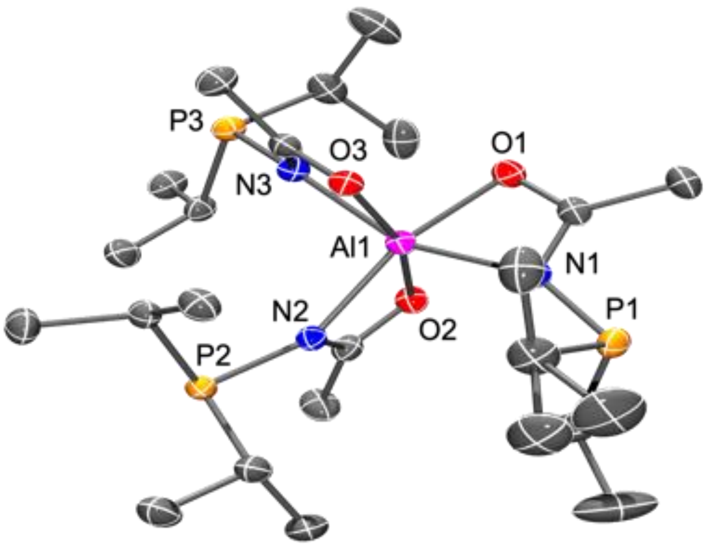

3

Figure S1. ORTEP drawings (50% probability ellipsoids) of **3** displaying selected labelling Hydrogen atoms omitted for clarity. Selected bond distances (Å) and angles (deg): Al1–N1, 2.0138(12); Al1–N2, 2.0491(11); Al1–N3, 2.0229(12); Al1–O1, 1.9190(9); Al1–O2, 1.8864(9); Al1–O3, 1.9033(9); O1–Al1–N1, 67.20(4); O2–Al1–N2, 67.60(4); O3–Al1–N3, 67.54(4).

X-Ray data collection, solution, and refinement for 4-Me (CCDC 2363381). A Leica M80 microscope was used to identify a suitable single colorless block-shaped crystal showing well defined faces with dimensions 0.25 × 0.09 × 0.04 mm^3 from a representative sample of crystals of the same habit. The crystal mounted on a nylon loop was then placed in a cold nitrogen stream (Oxford) maintained at T = 110 K. Crystal screening, unit cell determination, and data collection were carried out using a Bruker Quest (PHOTON III) diffractometer. The diffraction pattern was indexed and the total number of runs and images was based on the strategy calculation from the program APEX4.[6] Data was measured using ϕ and ω scans with MoK_α radiation. Data was collected to a maximum resolution of Θ = 27.527° (0.77 Å). The unit cell was refined using SAINT V8.40B on 9928 reflections, 19% of the observed reflections.[3] Integrated Intensity information for each reflection was obtained by reduction of data frames using SAINT V8.40B.[3] The final completeness is 99.90% out to 27.527° in Θ. SADABS-2016/2 was used for absorption correction.[4] wR_2(int) was 0.0950 before and 0.0661 after correction. The ratio of minimum to maximum transmission is 0.8991. The λ/2 correction factor is not present. The absorption coefficient μ of this material is 0.850 mm^{-1} at this wavelength (λ = 0.71073 Å) and the minimum and maximum transmissions are 0.381 and 0.423. Systematic reflection conditions and statistical tests of the data suggested the space group $P2_1/c$ and was confirmed by ShelXT 2018/2 structure solution program using dual methods.[5] The structure was refined by full matrix least squares minimization on $\boldsymbol{F^2}$ using version 2018/3 of XL.[5] All non-hydrogen atoms were refined anisotropically. Hydrogen atom positions were calculated geometrically and refined using the riding model.

X-Ray data collection, solution, and refinement for 4-H (CCDC 2363383). A Leica M80 microscope was used to identify a suitable single colorless needle-shaped crystal showing well defined faces with dimensions 0.26 × 0.05 × 0.03 mm^3 from a representative sample of crystals of the same habit. The crystal mounted on a nylon loop was then placed in a cold nitrogen stream (Oxford) maintained at T = 110 K. Crystal screening, unit cell determination, and data collection were carried out using a Bruker APEX-II CCD diffractometer. The diffraction pattern was indexed and the total number of runs and images was based on the strategy calculation from the program APEX 3.[6] Data was measured using *f* and *w* scans with Cu K_a radiation. Data was collected to a maximum resolution of Q = 70.38° (0.82 Å). The unit cell was refined using SAINT V8.40B on 4868 reflections, 15% of the observed reflections.[3] Integrated Intensity information for each reflection was obtained by reduction of data frames using SAINT V8.40B.[3] The final completeness is 99.61% out to 70.38° in Θ. SADABS-2016/2 was used for absorption correction.[4] wR_2(int) was 0.0950 before and 0.0622 after correction. The ratio of minimum to maximum transmission is 0.5382. The $\lambda/2$ correction factor is not present. The absorption coefficient μ of this material is 6.338 mm^{-1} at this wavelength (λ = 1.54178 Å) and the minimum and maximum transmissions are 0.173 and 0.322. Systematic reflection conditions and statistical tests of the data suggested the space group $P2_1/n$ and was confirmed by ShelXT 2018/2 structure solution program using dual methods.[5] The structure was refined by full matrix least squares minimization on F^2 using version of olex2.refine 1.5.[7] All non-hydrogen atoms were refined anisotropically. Most hydrogen atom positions were calculated geometrically and refined using the riding model, but some hydrogen atoms were refined freely.

V. NMR Spectra

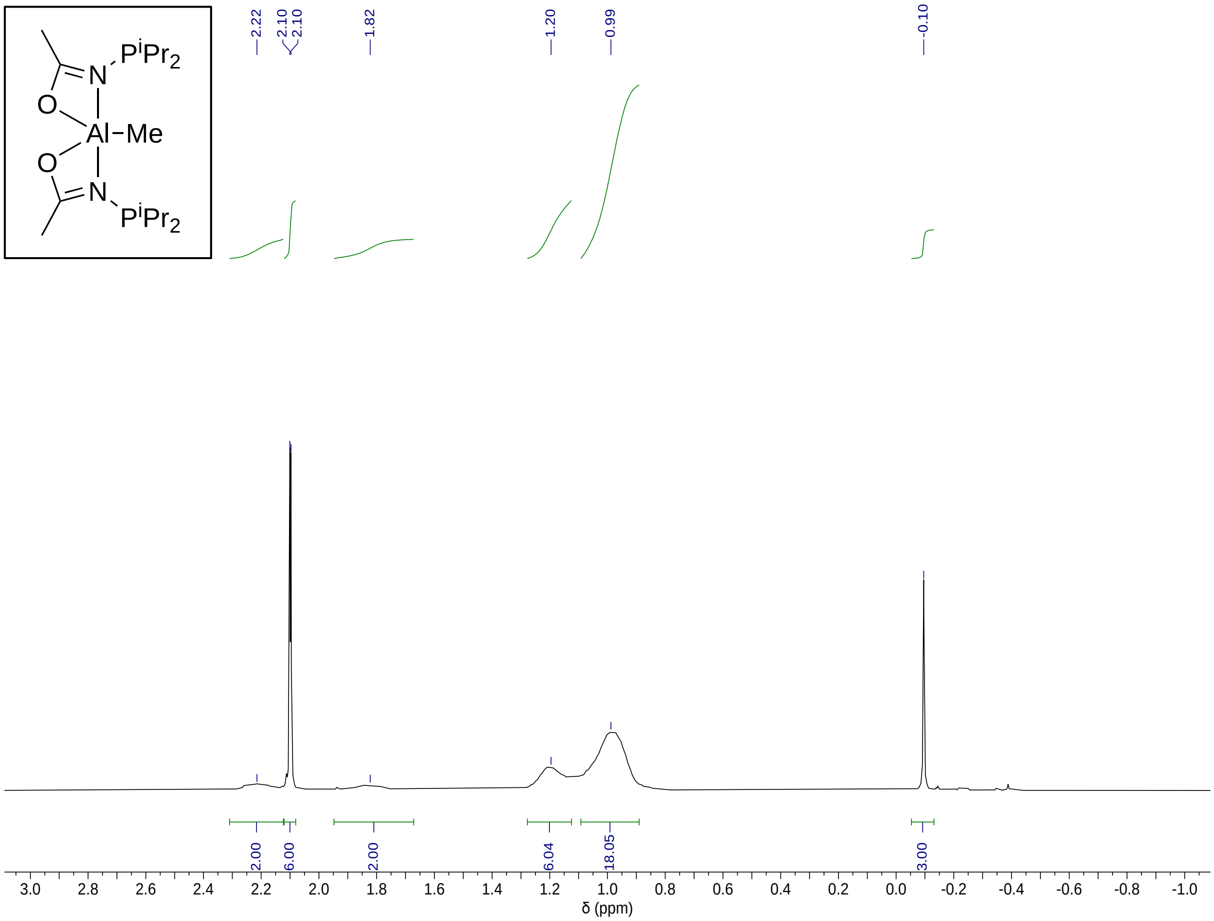

Figure S2. ^{1}H NMR (400 MHz, C_6D_6) spectrum of **2-Me**.

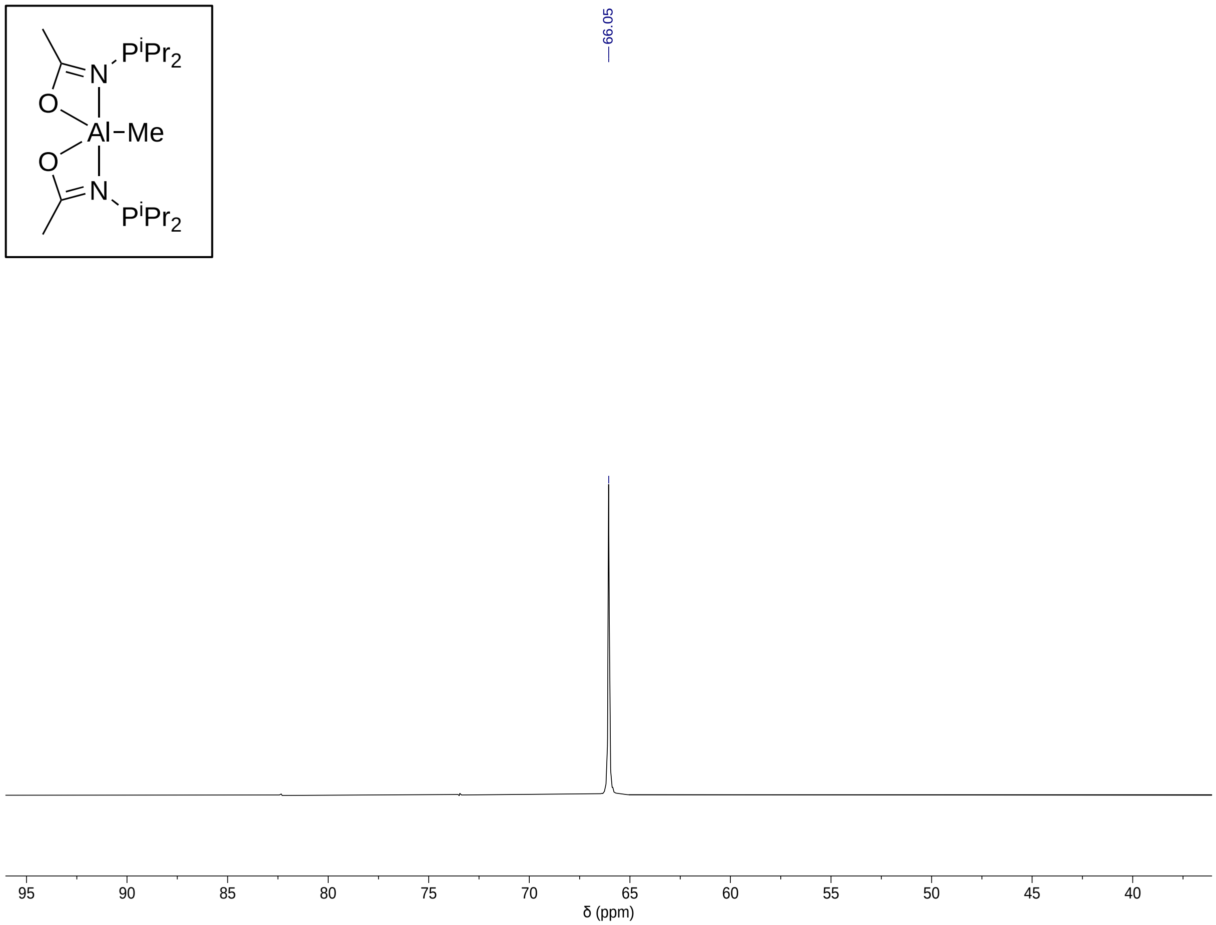

Figure S3. $^{31}P\{^{1}H\}$ NMR (162 MHz, C_6D_6) spectrum of **2-Me**.

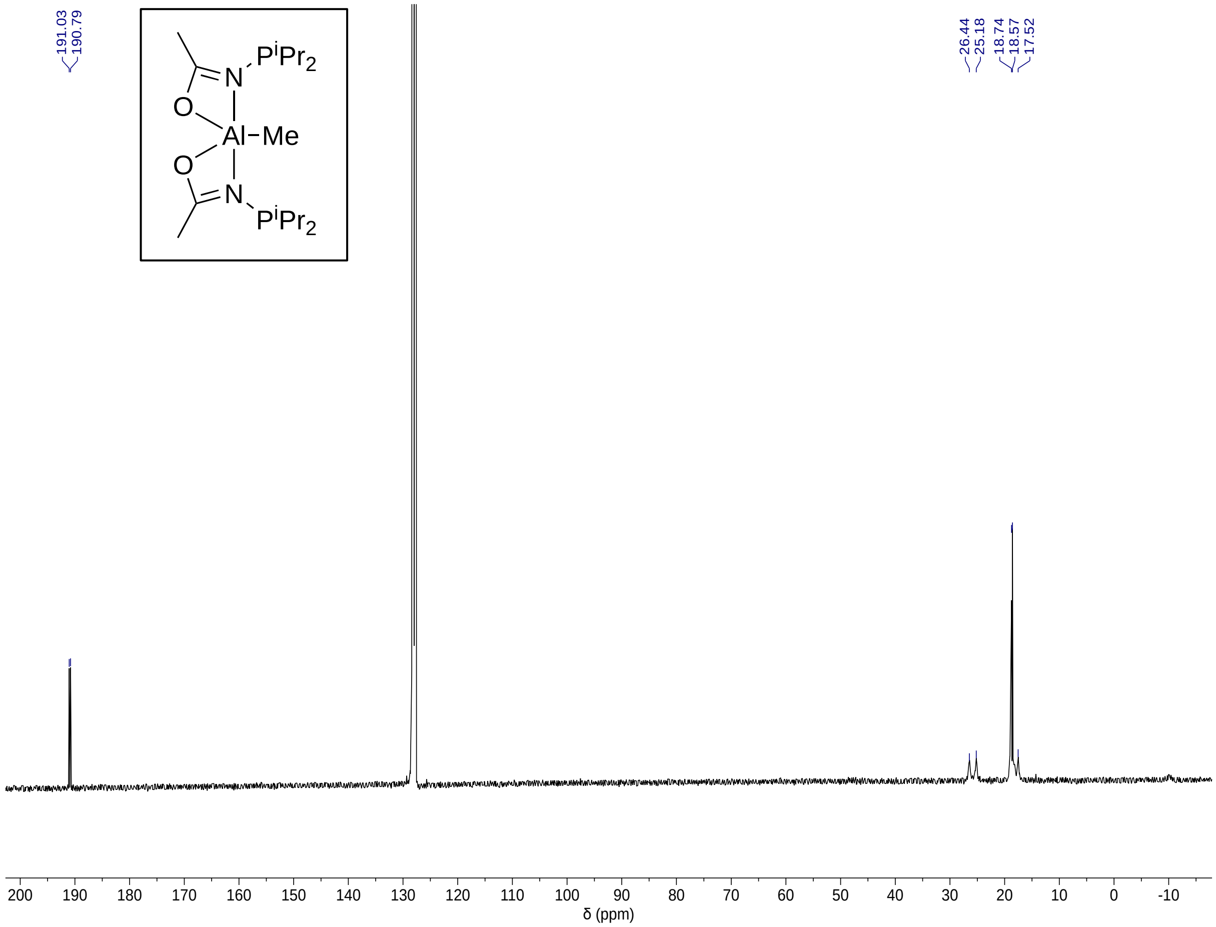

Figure S4. $^{13}C\{^1H\}$ NMR (101 MHz, C_6D_6) spectrum of **2-Me**.

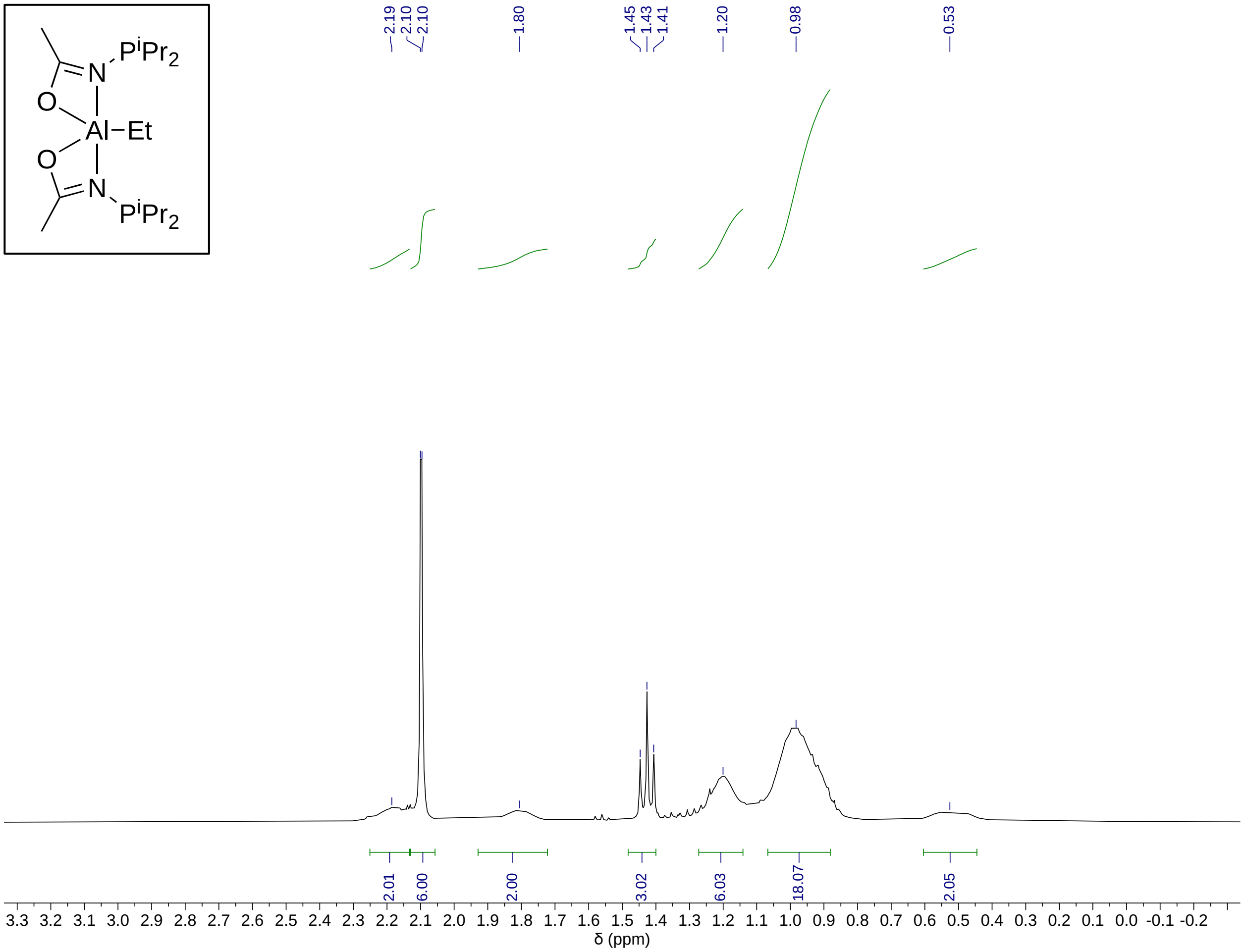

Figure S5. ^{1}H NMR (400 MHz, C_6D_6) spectrum of **2-Et**.

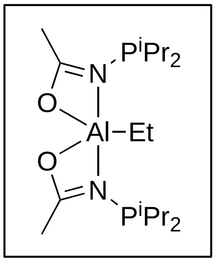

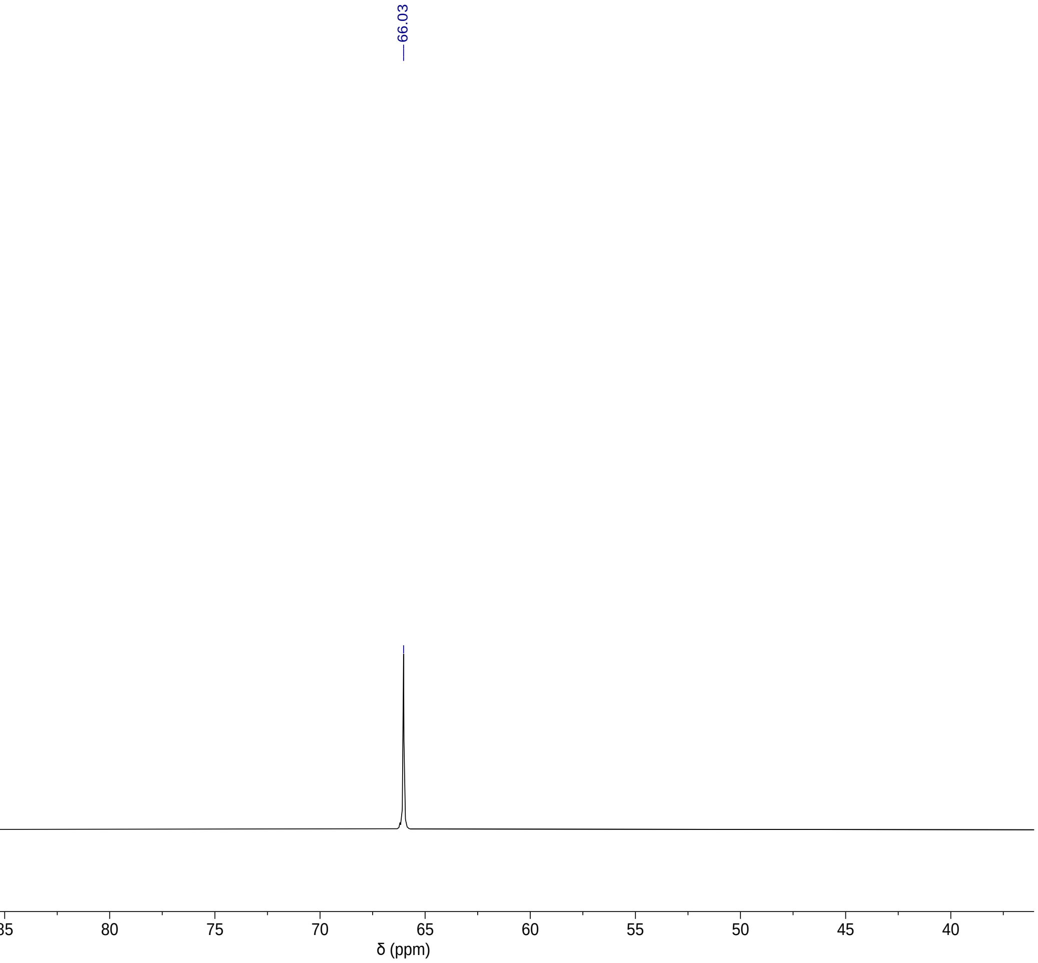

Figure S6. $^{31}P\{^{1}H\}$ NMR (162 MHz, C_6D_6) spectrum of **2-Et**.

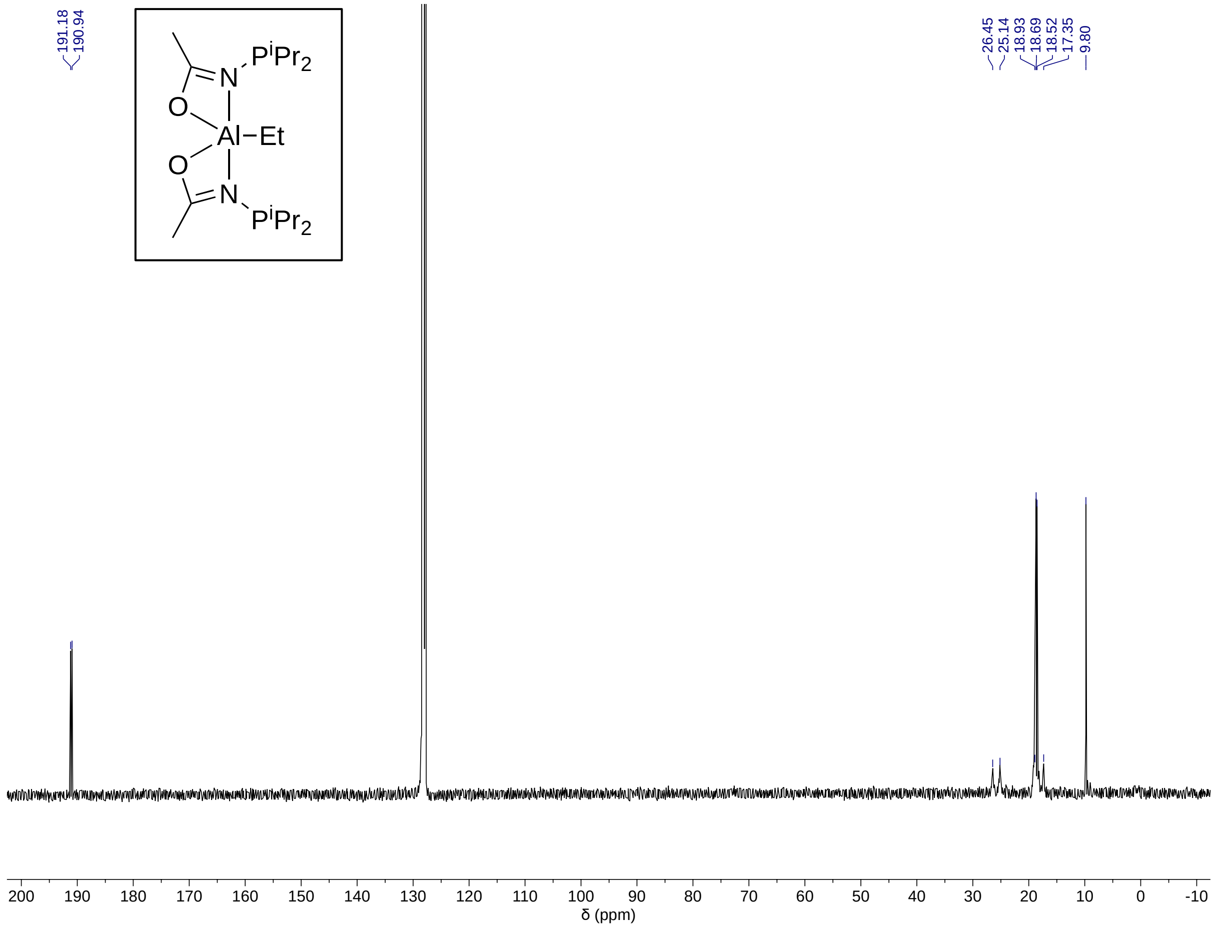

Figure S7. $^{13}C\{^{1}H\}$ NMR (101 MHz, C_6D_6) spectrum of **2-Et**.

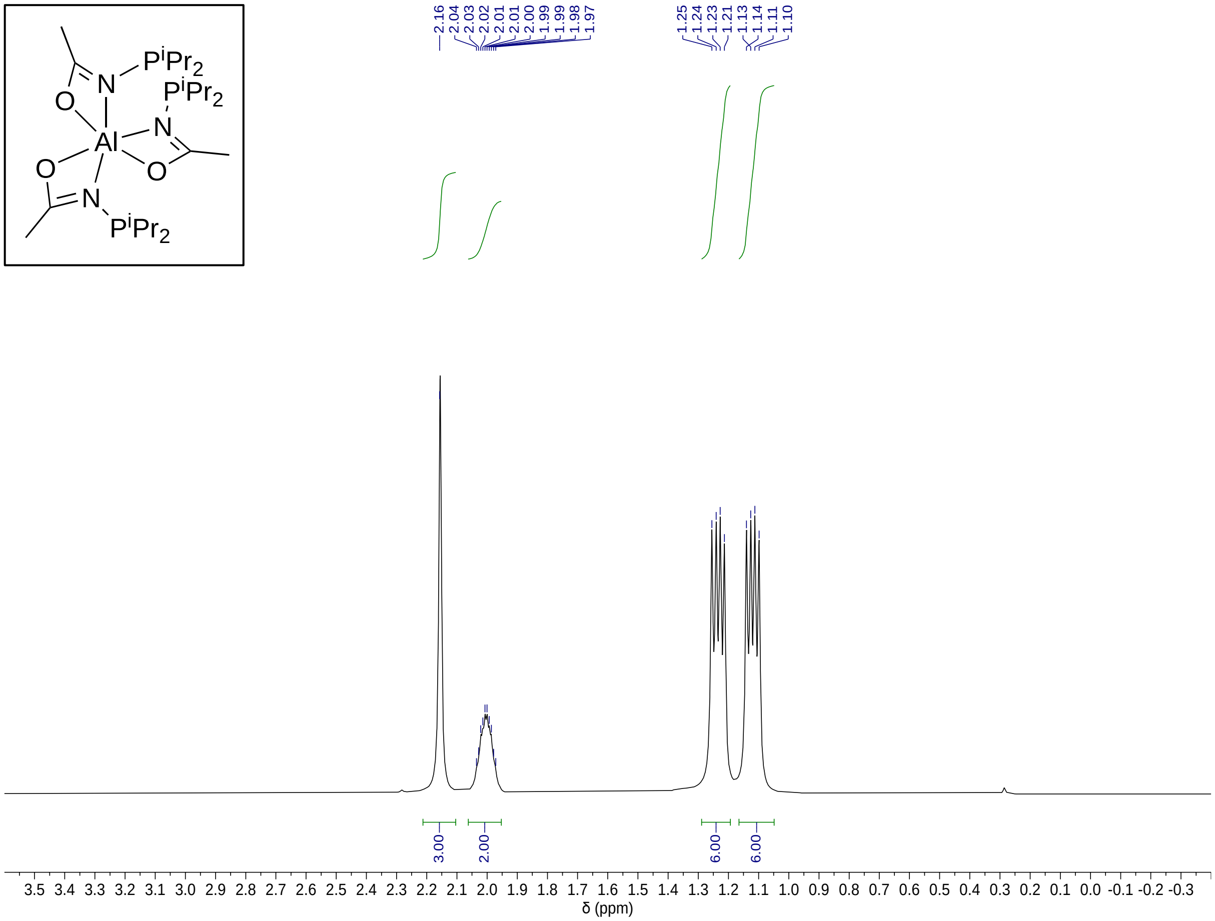

Figure S8. ^{1}H NMR (500 MHz, C_6D_6) spectrum of **3**.

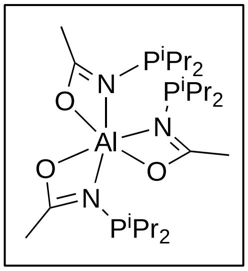

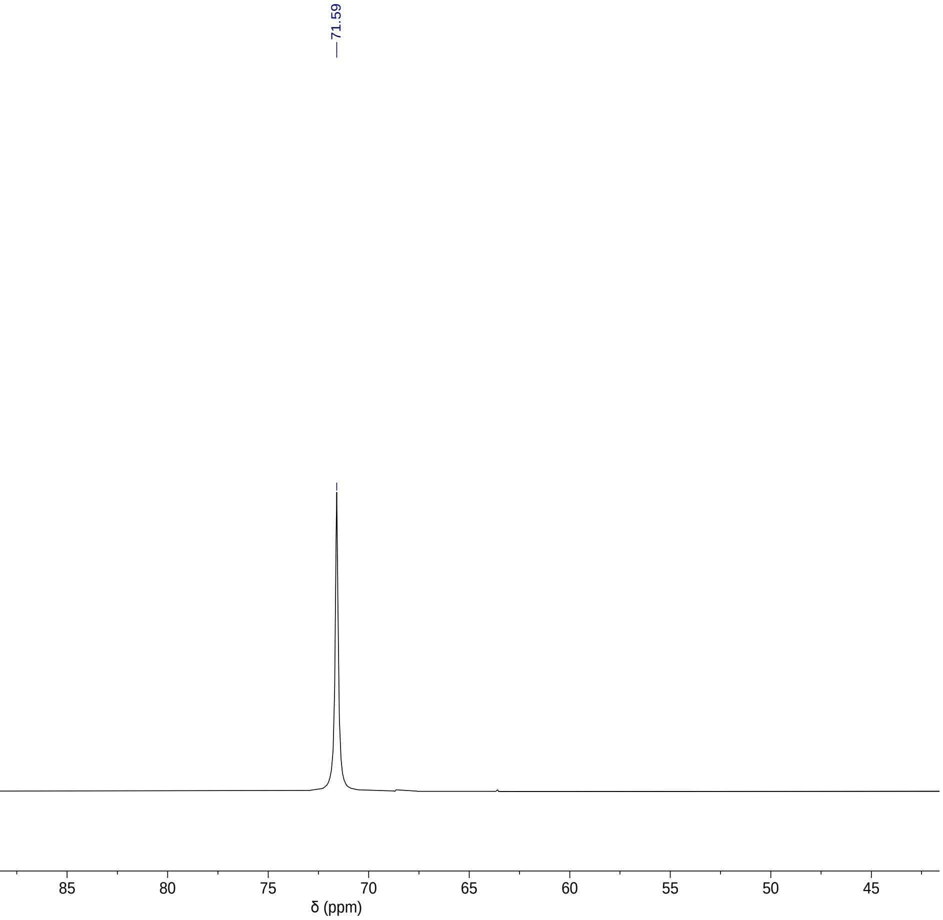

Figure S9. $^{31}P\{^{1}H\}$ NMR (202 MHz, C_6D_6) spectrum of **3**.

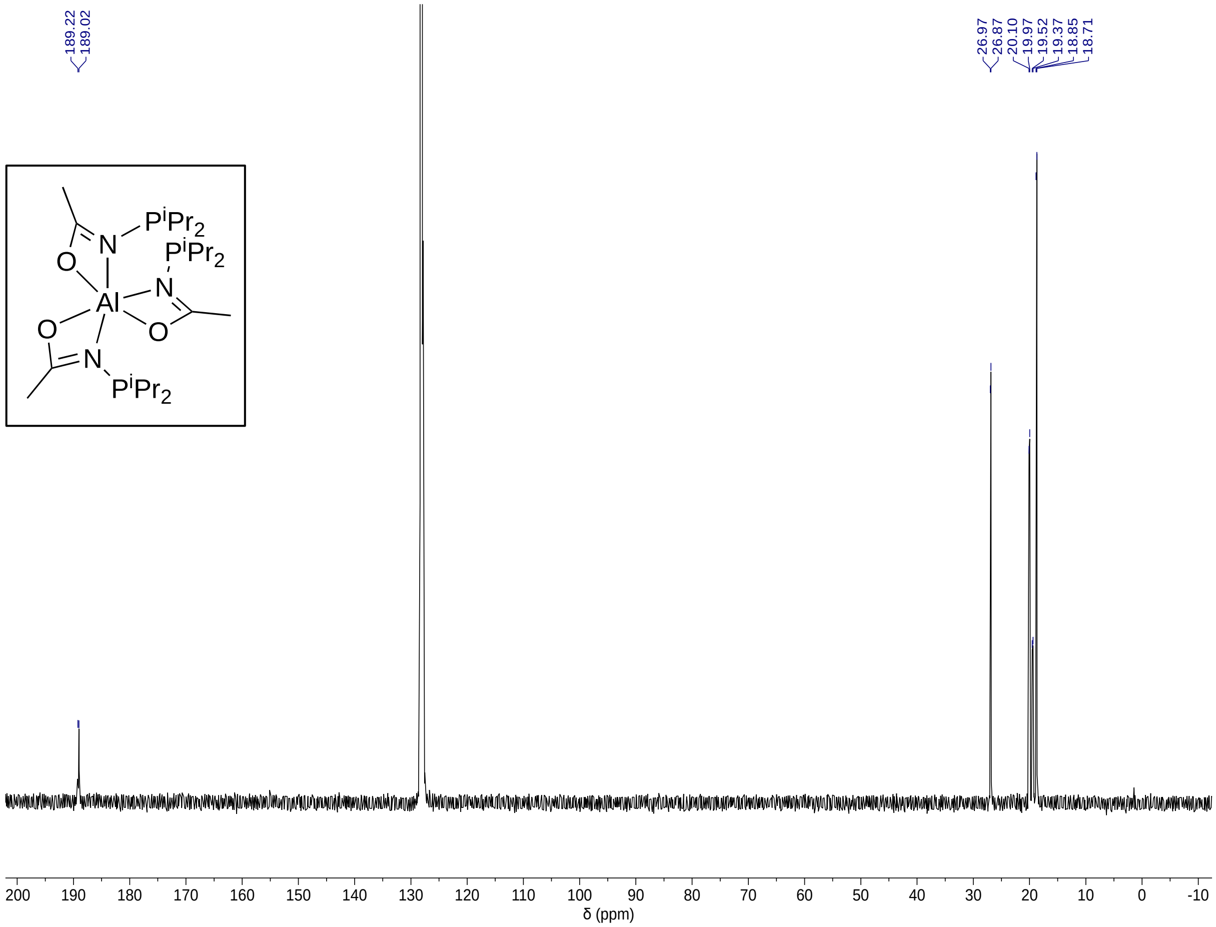

Figure S10. $^{13}C\{^1H\}$ NMR (126 MHz, C_6D_6) spectrum of **3**.

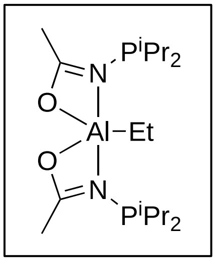

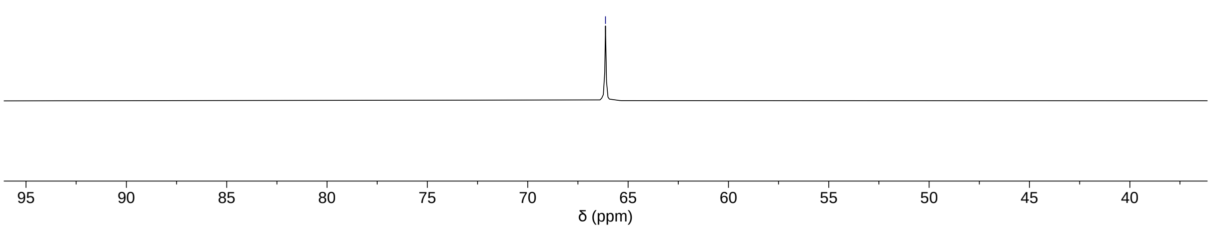

Figure S11. $^{31}P\{^{1}H\}$ NMR (162 MHz, C_6D_6) spectrum of the comproportionation of **3** with triethylaluminum.

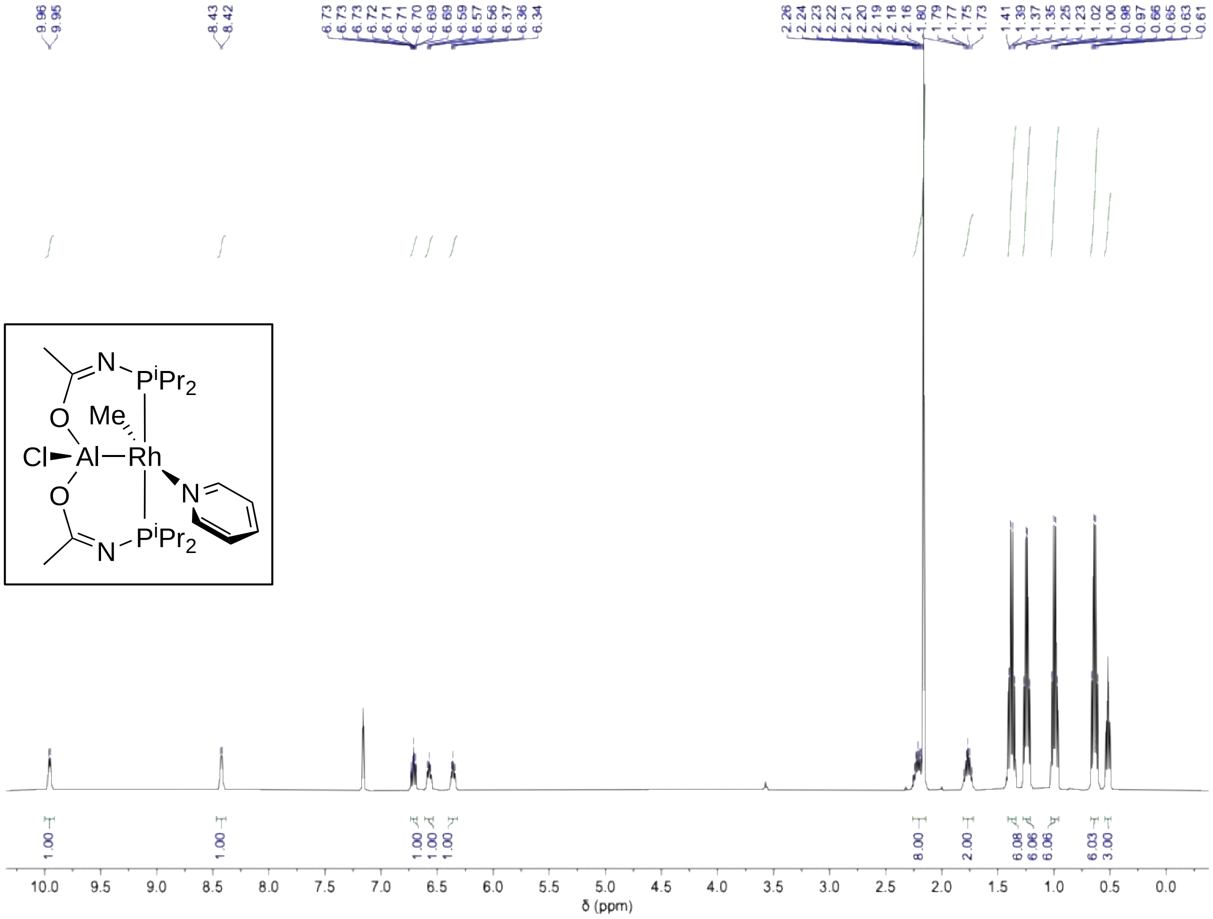

Figure S12. ^{1}H NMR (400 MHz, C_6D_6) spectrum of **4-Me**.

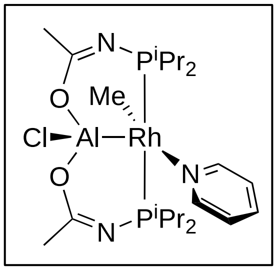

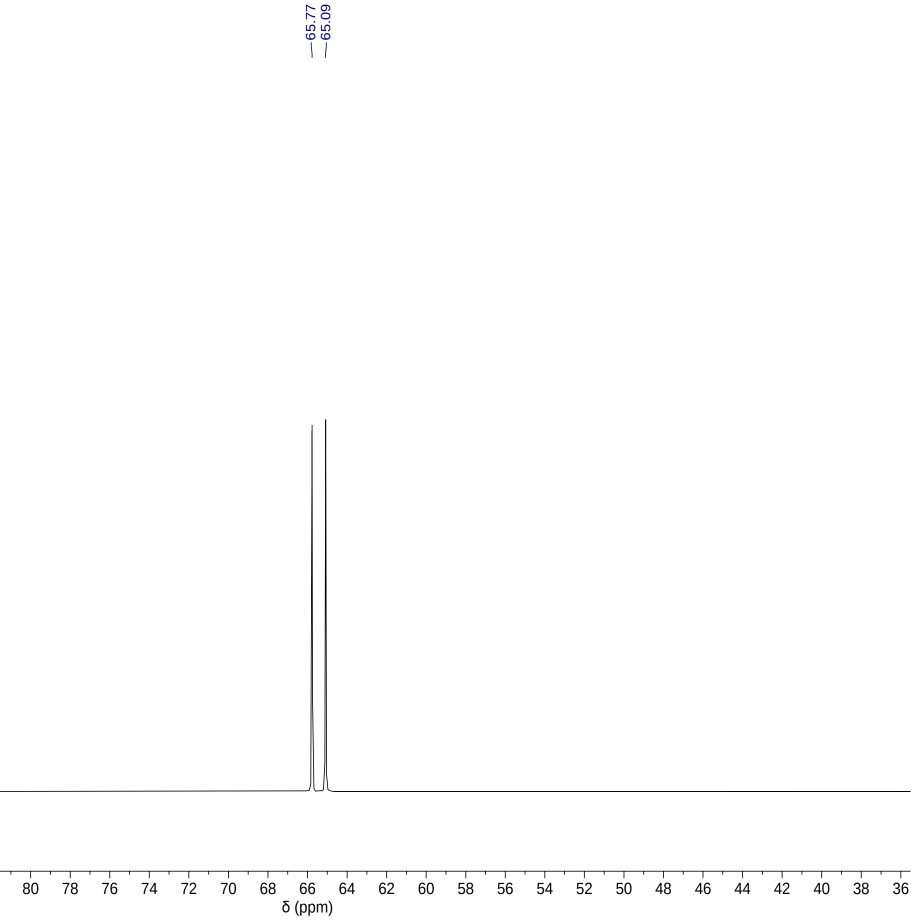

Figure S13. $^{31}P\{^1H\}$ NMR (162 MHz, C_6D_6) spectrum of **4-Me**.

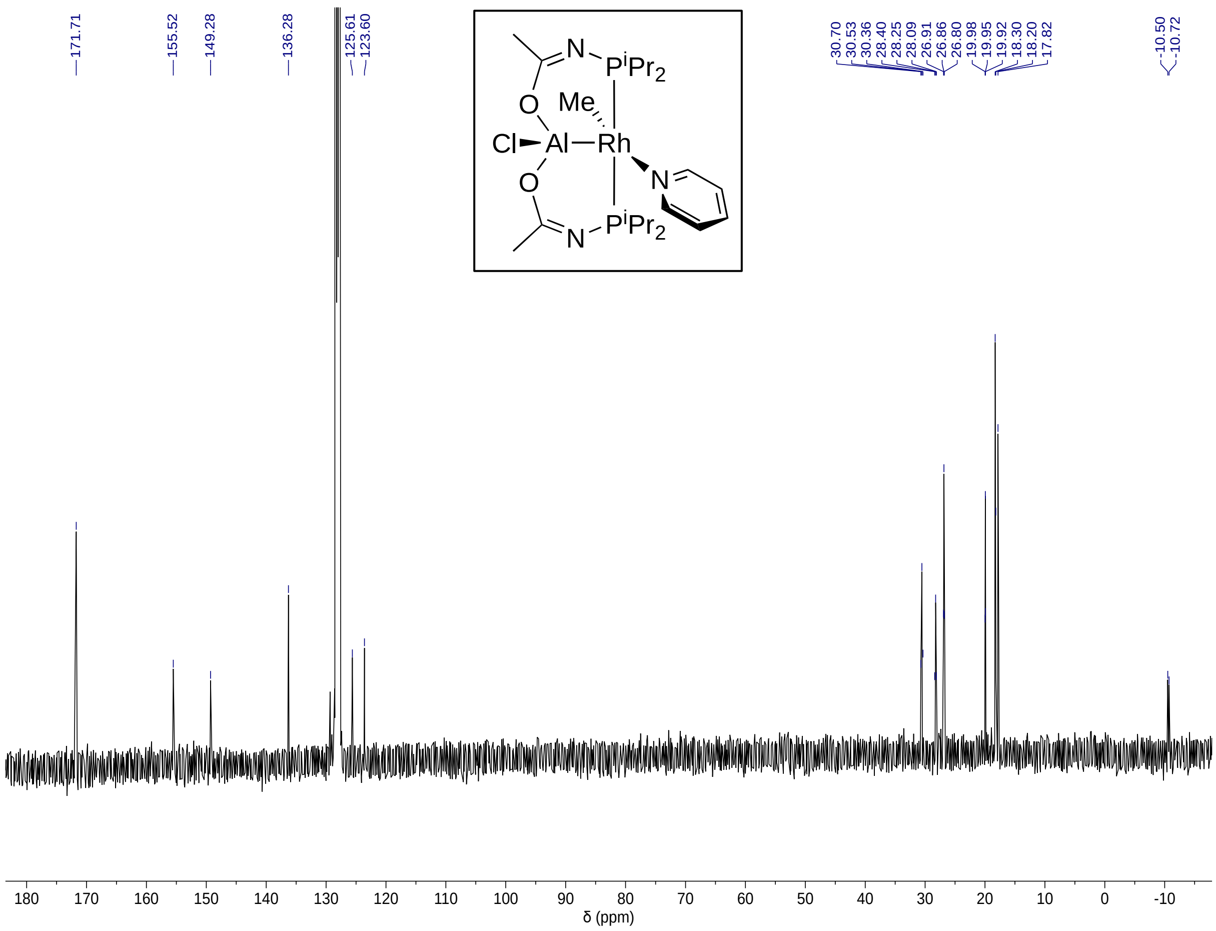

Figure S14. $^{13}C\{^{1}H\}$ NMR (101 MHz, C_6D_6) spectrum of **4-Me**.

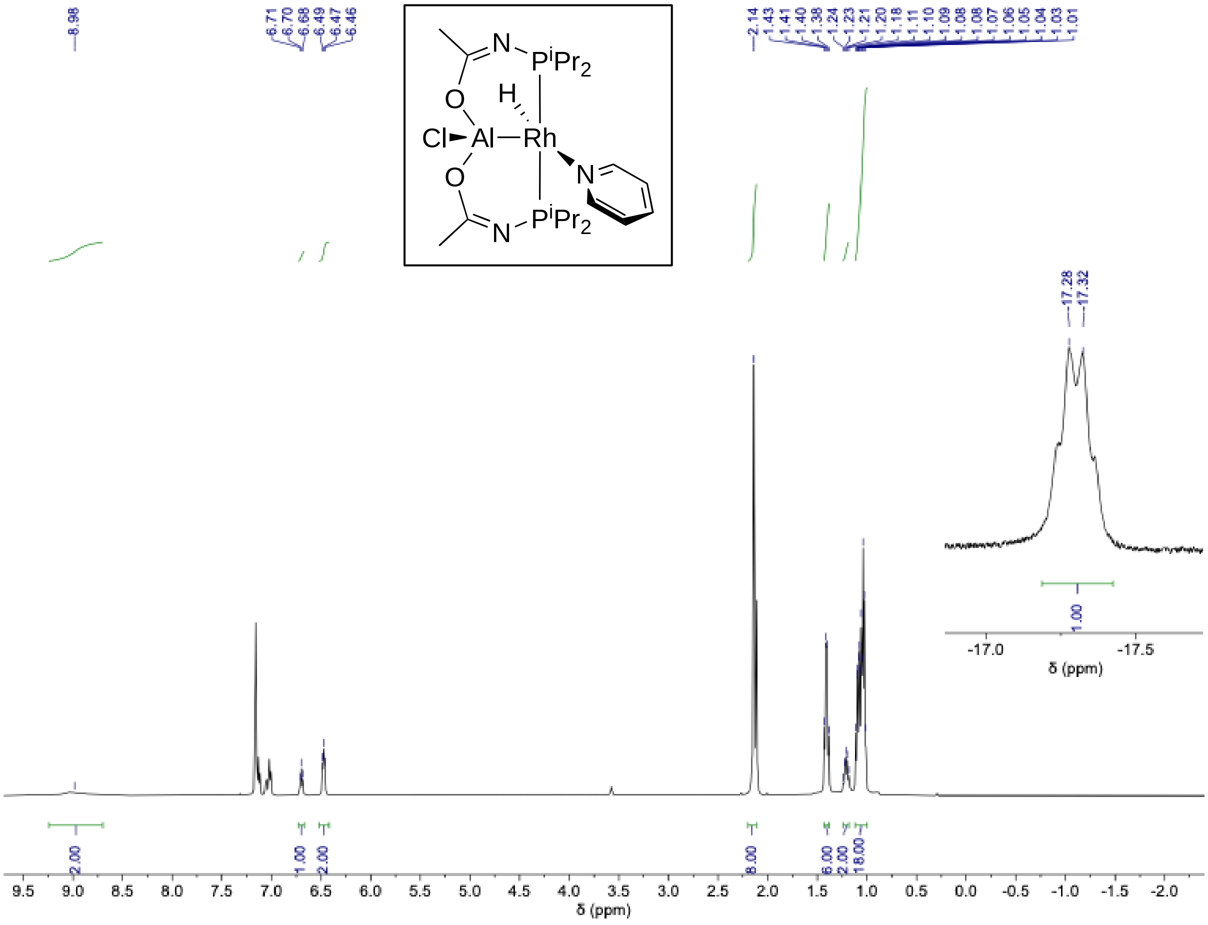

Figure S15. ^{1}H NMR (500 MHz, C_6D_6) spectrum of **4-H**. Sample contains residual THF and toluene.

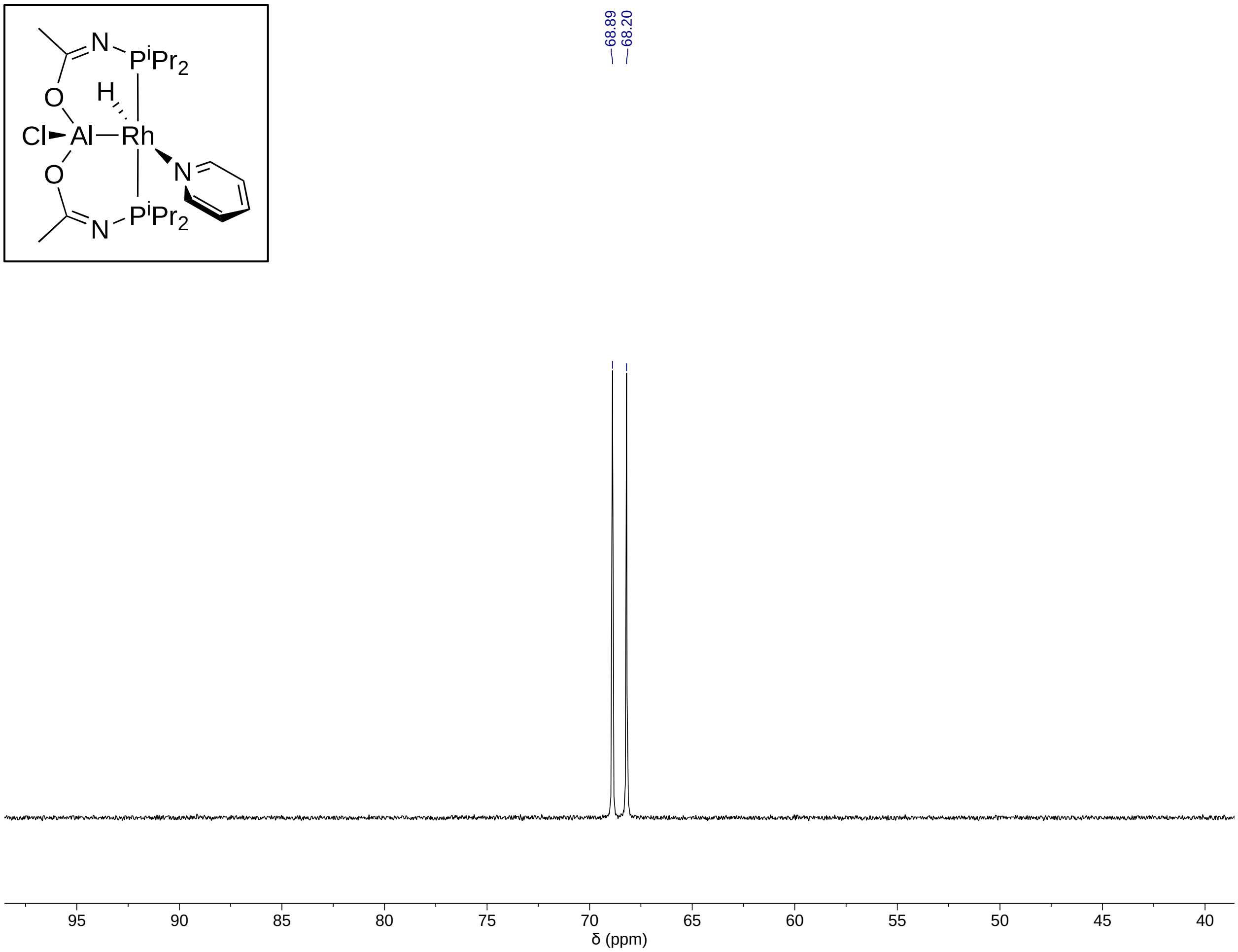

Figure S16. $^{31}P\{^{1}H\}$ NMR (202 MHz, C_6D_6) spectrum of **4-H**.

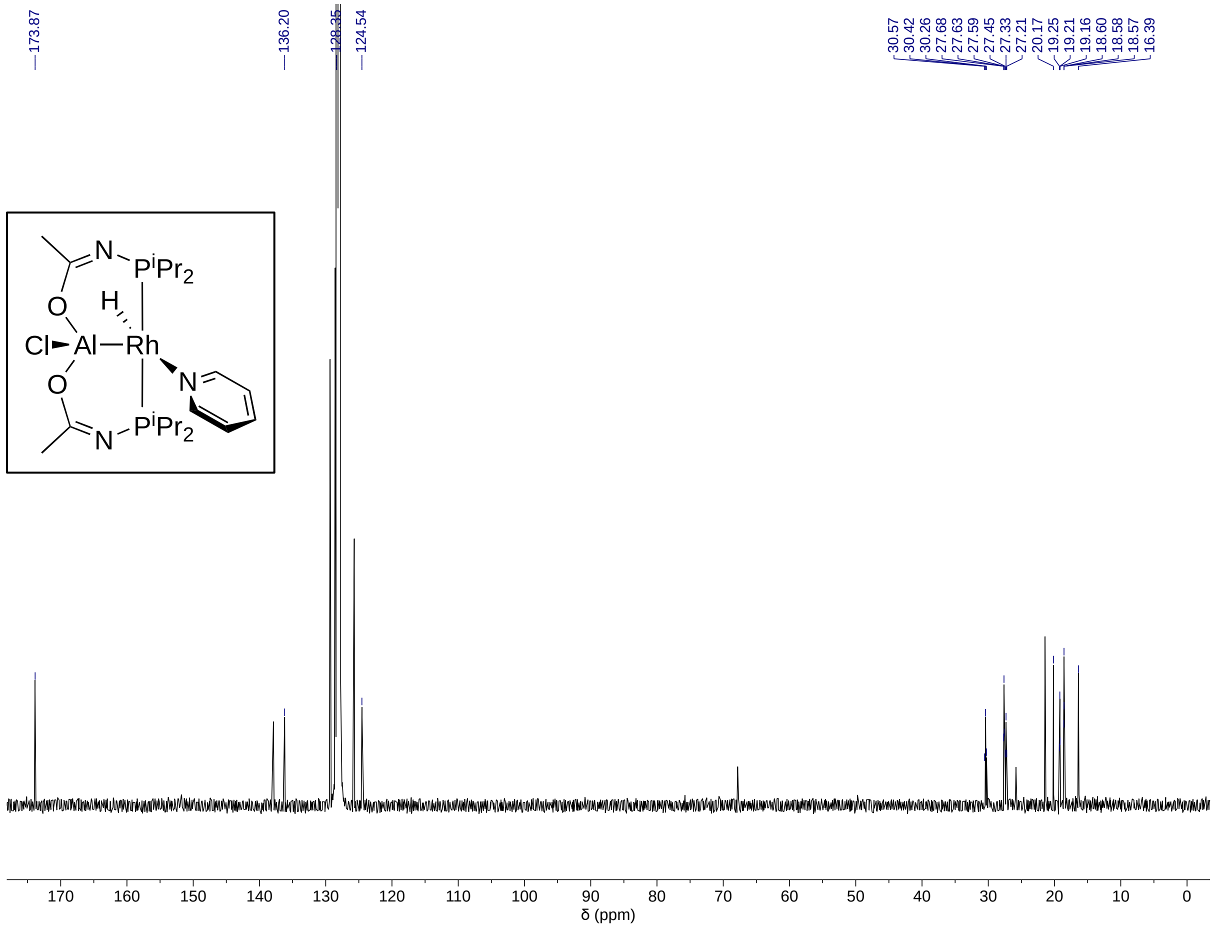

Figure S17. $^{13}C\{^1H\}$ NMR (126 MHz, C_6D_6) spectrum of **4-H**. Sample contains residual THF and toluene.

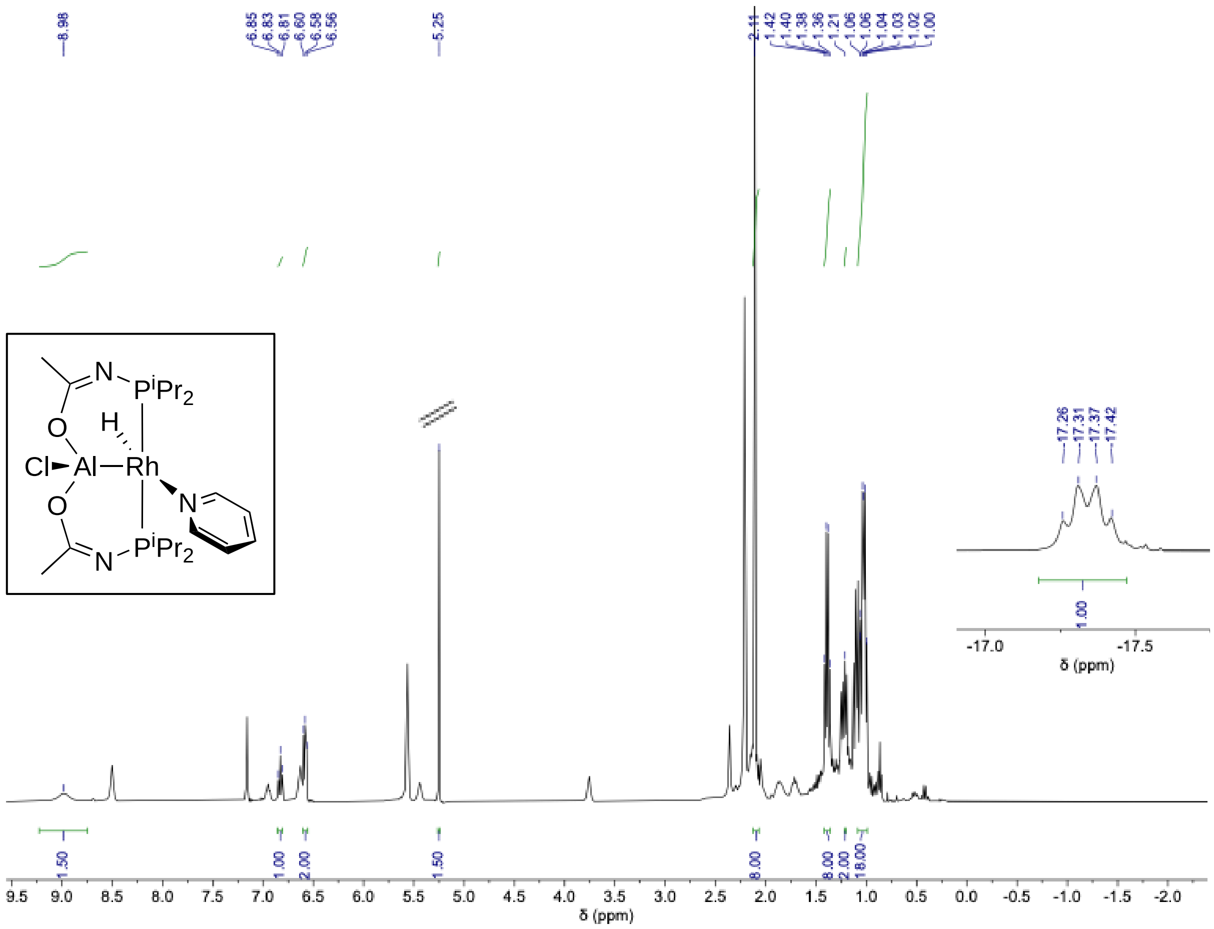

Figure S18. ^{1}H NMR (400 MHz, C_6D_6) spectrum of the *in situ* observation of ethylene during the metalation of **2-Et.**

VI. SI References

[1] Agostinho, M.; Rosa, V.; Avilés, T.; Welter, R.; Braunstein, P. Synthesis and characterization of Co and Ni complexes stabilized by keto- and acetamide-derived P,O-type phosphine ligands. *Dalton Trans.* **2009**, *18*, 814-822.

[2] Giordano, G.; Crabtree, R. H.; Heintz, R. M.; Forster, D.; Morris, D. E. Di-μ-Chloro-Bis(η^4-1,5-Cyclooctadiene) Dirhodium(I). *Inorg. Synth.* **1979**, *19*, 218-220.

[3] (a) Bruker, SAINT, V8.38A, Bruker AXS Inc., 5465 East Cheryl Parkway, Madison, WI 53711-5373 USA. (b) Bruker, SAINT, V8.40B, Bruker AXS Inc., 5465 East Cheryl Parkway, Madison, WI 53711-5373 USA.

[4] SADABS, Sheldrick, G.M. "*Program for Absorption Correction of Area Detector Frames*", BRUKER AXS Inc., 5465 East Cheryl Parkway, Madison, WI 53711-5373 USA.

[5] (a) G. M. Sheldrick, *Acta Cryst.*, 2008, **A64**, 112-122. (b) G. M. Sheldrick, *Acta Cryst.*, 2015, **A71**, 3-8. (c) G. M. Sheldrick, *Acta Cryst.*, 2015, **C71**, 3-8. (d) XT, XS, BRUKER AXS Inc., 5465 East Cheryl Parkway, Madison, WI 53711-5373 USA.

[6] (a) APEX3 "Program for Data Collection on Area Detectors" BRUKER AXS Inc., 5465 East Cheryl Parkway, Madison, WI 53711-5373 USA. (b) APEX4 "Program for Data Collection on Area Detectors" BRUKER AXS Inc., 5465 East Cheryl Parkway, Madison, WI 53711-5373 USA.

[7] Bourhis, L. J.; Dolomanov, O. V.; Gildea, R. J.; Howard, J. A. K.; Puschmann, H., The Anatomy of a Comprehensive Constrained, Restrained, Refinement Program for the Modern Computing Environment - Olex2 Disected, *Acta Cryst. A*, 2015, **A71**, 59-71.
